# Effect of variable transmission rate on the dynamics of HIV in sub-Saharan Africa

**DOI:** 10.1186/1471-2334-11-216

**Published:** 2011-08-11

**Authors:** Diego F Cuadros, Philip H Crowley, Ben Augustine, Sarah L Stewart, Gisela García-Ramos

**Affiliations:** 1 Department of Biology, University of Kentucky, Lexington, KY, USA

## Abstract

**Background:**

The cause of the high HIV prevalence in sub-Saharan Africa is incompletely understood, with heterosexual penile-vaginal transmission proposed as the main mechanism. Heterosexual HIV transmission has been estimated to have a very low probability; but effects of cofactors that vary in space and time may substantially alter this pattern.

**Methods:**

To test the effect of individual variation in the HIV infectiousness generated by co-infection, we developed and analyzed a mathematical sexual network model that simulates the behavioral components of a population from Malawi, as well as the dynamics of HIV and the co-infection effect caused by other infectious diseases, including herpes simplex virus type-2, gonorrhea, syphilis and malaria.

**Results:**

The analysis shows that without the amplification effect caused by co-infection, no epidemic is generated, and HIV prevalence decreases to extinction. But the model indicates that an epidemic can be generated by the amplification effect on HIV transmission caused by co-infection.

**Conclusion:**

The simulated sexual network demonstrated that a single value for HIV infectivity fails to describe the dynamics of the epidemic. Regardless of the low probability of heterosexual transmission per sexual contact, the inclusion of individual variation generated by transient but repeated increases in HIV viral load associated with co-infections may provide a biological basis for the accelerated spread of HIV in sub-Saharan Africa. Moreover, our work raises the possibility that the natural history of HIV in sub-Saharan Africa cannot be fully understood if individual variation in infectiousness is neglected.

## Background

The cause of the high HIV-1 prevalence in sub-Saharan Africa is incompletely understood [1-3]. Unlike HIV in the US and Europe, which seems concentrated among injection drug users and men who have sex with men [1,2,4], the epidemic in Africa is more widely distributed across the general population, with heterosexual penile-vaginal transmission proposed as the main mechanism [4-7].

Mathematical models are powerful tools in epidemiology: they can facilitate understanding of the interplay between the variables that determine the course of infection within an individual and the variables that control the pattern of infections within communities of people. But mathematical modeling studies that attempt to reproduce the observed HIV epidemic curve in sub-Saharan Africa are often criticized for using per-contact and per-partnership heterosexual transmission efficiencies that are improbably high [8,9]. For example in the calculation of per-partner rate of transmission, behavioral parameters such as number of sexual partners per year and number of sexual contacts per partner may be overestimated by assuming levels of promiscuity in African societies that are too high [8].

Unlike more traditional epidemiological approaches that focus strictly either on individuals or on populations, sexual networks are based on the dynamics of the sexual links (connections between nodes = between individuals) and the topology of the linkage in a group [10,11]. Sexual networks have multiple advantages for characterizing individual heterogeneity of sexual behavior. This approach to understanding the spread of a sexually transmitted infection (STI) has focused attention on the properties of the frequency distribution of sexual partner number. In sexual networks, partner number is the node degree, the number of sexual links that each node (individual) has to others [12]. Thus network studies mainly focus on the distribution of node degree, which can be characterized by the data [13].

Network models also focus on other components of the network structure that cannot be described from the observation of individual nodes alone. The degree distribution is only one example of an aggregate statistics obtained by the study of the individual properties within the network. For the calculation of other statistics, such as the level of clustering, it would be necessary to observe larger fragments of the network [14]. Clustering measures focus on describing both the connections from focal nodes and the connections made by its neighbors. In particular, high levels of clustering may reduce the rate of spread of an infectious disease [15].

The typically high skew of sexual degree distributions has suggested that sexual networks may follow a power law (scale-free) distribution [16,17]. Power law distributions are characterized by many nodes with only one or few connections but also a few nodes with many more connections, generating a high contact variance. The high variance observed in large populations that follow the power law distributions implies that even very low transmission rates are consistent with disease spread [11,18].

Most of the studies that have attempted to describe sexual behavior in Africa have found that the power law distribution does not adequately fit the data. Instead, fixed rate models such as the negative binomial model, which is a generalization of the Poisson model, appear to fit the degree distribution best [12,13]. In the negative binomial model, the propensities of individuals to form connections are estimated from a gamma distribution. This approach, with its lower variance in connectedness among nodes, raises the possibility that the infectivity of HIV may be an important determinant of the epidemic in sub-Saharan Africa [10,18].

Yet this conclusion is inconsistent with the low probability of heterosexual HIV transmission, estimated to be ~ 1/300 per coital act in low income countries [19,20]. Moreover, studies that attempted to estimate the probability of HIV transmission per sexual contact have found that the Bernoulli model accurately estimates the per-partner probability of HIV transmission but does not seem to correlate with the number of sex acts and thus fails to estimate the per sexual contact probability of transmission [21]. It has been suggested that the constant transmission probability in the Bernoulli model may be the problem: variability of infectiousness among individuals and over time, such as may arise from important transmission cofactors, may be essential for a realistic representation of HIV transmission [21-23].

Despite the low probability of heterosexual penile-vaginal transmission per sexual contact, some studies have demonstrated that the risk of HIV transmission can be strongly correlated with variation in blood viral burden [24-26]. The most relevant finding from these studies is that infectiousness can be directly correlated with the concentration of HIV-RNA in blood, which indicates shedding of the virus into genital track secretions.

In a pioneering study attempting to correlate the viral load and the transmission of the virus, Quinn et al. [27] measured the HIV-RNA load in the blood of more than 15000 subjects. They found that the virus was hardly ever transmitted by infected subjects with less than 1500 copies of HIV-RNA/ml, whereas individuals with more than 50 000 copies infected their sexual partners at a rate of 23 per 100 person-years over 30 months.

A similar study conducted with discordant couples for HIV status in Uganda showed the existence of a strong correlation between HIV plasma viral load and HIV transmission rates [28]. The Uganda study indicated that a ten-fold increment in viral load could increase the risk of HIV transmission per sexual contact 2.45-fold (95% confidence interval 1.85-3.26). They pointed out that although blood and semen reside in separate biological compartments, blood viral burden can be correlated with viral burden in semen.

Growing evidence suggests the existence of additional biological factors that cause variations in the viral load. The viral set point is actually not constant and may be perturbed by reactivations of the immune system, such as those resulting from the invasion of other pathogens [29]. Changes in the host immune response may account for variations in the viral load that could make the host more infectious and increase the risk of transmission.

The average African host is usually exposed to numerous bacterial, viral and parasitic infections. Of special importance is the very high prevalence of STIs, particularly genital ulcerations caused by herpes simplex virus type 2 (HSV-2) [29]. The existence of a synergistic relationship between HIV and HSV-2 has been strongly suggested by many observational and biological studies in which HSV-2 has been implicated as a biological cofactor for the acquisition and transmission of HIV [30,31].

The rapid spread of HIV as a sexually transmitted disease is exceeded by that of HSV-2 [32]. The prevalence of HSV-2, which may be as high as 75% among women in parts of sub-Saharan Africa [33], has reached a prevalence of up to 90% in HIV-positive persons [31].

While bacterial STIs such as gonorrhea and syphilis, which also amplify the risk of HIV transmission [34], tend to be concentrated in high risk groups [35], the biological characteristics of HSV-2 allow this virus to be sustainable at high levels in the general population, as observed in sub-Saharan Africa [36]. Consequently, as the HIV epidemic reaches the general population, the epidemiological overlap between HSV-2 and HIV is considerably larger than any other STI.

The ulcers caused by HSV-2 contain substantial numbers of CD4+ lymphocytes, the target cell for HIV, and therefore are likely to facilitate the acquisition of HIV in HIV-negative individuals [37]. Additionally, the high levels of HIV-RNA in herpetic lesions from dually infected patients [38] may be explained by studies *in vitro *demonstrating that HSV-2 increases HIV transcription, which supports the higher infectivity in co-infected individuals. Population-based studies have also demonstrated that HIV-RNA levels can increase during active HSV-2 infection [39], and suppression of HSV-2 with acyclovir was associated with a measurable decrease on the HIV-RNA levels [40].

The enhanced HIV infectivity caused by HSV-2 co-infection has also been corroborated by population-based studies suggesting a relative risk of three to five-fold of HIV transmission from co-infected individuals compared to HSV-2 seronegative persons [36,41,42]. These data suggest that HSV-2 may be playing a key role fueling the HIV epidemic in sub-Saharan Africa [43].

The activation of the immune system, however, is not only produced by STIs. Parasitic infections such as helminth infections, leishmaniasis and malaria might produce a strong response from the immune system and consequently generate similar effects on the replication of the virus in HIV co-infected individuals [29,44-47]. The geographical overlap observed between malaria and HIV infections has suggested a possible interaction influencing HIV transmission in some countries of sub-Saharan Africa. Malaria occurs throughout the tropical world, where it remains one of the most prevalent infectious diseases, with an estimated 300 million cases per year [48].

The evidence of an interaction between malaria and HIV comes from various sources. Several *in vitro *studies have found that malaria antigens significantly enhanced HIV-1 replication [44-46,49]. Additionally, population-based studies conducted with HIV-1 infected adults have indicated that the HIV-1 RNA concentration almost doubled between baseline (96,215 copies per ml) and those co-infected with malaria (168,901 copies per ml). The authors concluded that HIV-positive individuals co-infected with malaria had a significantly increased viral load and possibly increased infection transmission [45].

Based on the evidence previously mentioned, this study examines the limitations of the view that the level of the HIV epidemic in sub-Saharan Africa could be explained merely by a constant probability of transmission. We suspected that disregarding the variation across individuals in HIV infectivity would fail to replicate the HIV epidemic observed in a sexual network from sub-Saharan Africa. Instead, we predicted that individual and temporal variations in HIV transmission generated by biological factors such as co-infections with other infectious diseases could explain the severity of the HIV epidemic.

## Methods

With the aim of testing the effect of temporal and individual variation on HIV transmission generated by co-infection, we developed a dynamic sexual network model [15]. Partnership acquisition process relevant to HIV infections is too complex to be adequately captured by a static degree distribution [50]. Other nodal attributes such as gender, age and marital status are also of fundamental importance, as are the dynamics of the linkages themselves. To include these characteristics, we used Monte Carlo simulations to depict a dynamic sexual network with given nodal and structural characteristics, where links between nodes are formed and dissolved according to estimated parameters. The model incorporates the dynamic of the behavioral components of the population, as well as the dynamics of HIV and the co-infection effect on the HIV transmission caused by other infectious diseases, including HSV-2, gonorrhea, syphilis and malaria, along with the spread of HIV infections caused by commercial sex. We used data from studies in Malawi when available as an example of a generalized HIV epidemic [51-53].

### Model structure

A stochastic, individual-based sexual network model was created to simulate disease dynamics using the MATLAB^® ^computing language version 7 [54]. The model was divided in two main modules: a behavioral module and an epidemiological module.

Sexual partnerships were assumed to be exclusively heterosexual, and two types of partnerships, distinguished by duration, were considered. The population size remained constant, with individuals maturing into the network to offset those who die or mature out of the network. In accordance with the highest resolution of relevant data, a monthly time step was used. With this model, the effects of network structure on disease transmission, relationship type, and co-infection with other infectious diseases were evaluated.

For the estimation of the main parameters of the sexual network, data were used from a study of Malawi by the University of Pennsylvania Population Study Center and called "The Malawi Social Network Project" [51], as well as data from the Demographic and Health Survey (DHS) database from Malawi [55]. The study was conducted in three districts of Malawi, and the sampling strategy is explained elsewhere [52,53]. The study focuses on the description of the sexual behavior in the Malawi population, where the more important characteristics such as age distribution, number of sexual partners per year, type of relationship, duration of the relationship and age mixing patterns of marriage were derived. Additional file 1, Table S1 lists the key assumptions of the behavioral module.

Equal numbers of individuals of each sex were created and assigned an age and node degree (maximum number of partners per year). Consequently, individual age was used to determine when individuals should be removed from the sexual network and was the basis for other age-specific traits.

The epidemiological module was subdivided into two steps, the spread of the infections, and the progression and recovery of each infection. We selected gonorrhea and syphilis as examples of bacterial STIs concentrated in the high-risk (core) groups based on the amplification effect on HIV transmission, and their relevance in terms of prevalence in the Malawi population [56,57]. The dynamics of these infections are well known, and the effect of each infection on the transmission of HIV has been determined.

We also included two infections with high prevalence in the general population: herpes simplex virus type 2 (HSV-2) and malaria. The chronic nature of HSV-2 and its relatively high transmission efficiency make it sustainable in the general population. HSV-2 reactivations increase HIV transcription [58], which in turn generates an increase in the HIV plasma viral load [59] and supports higher HIV infectivity in dually infected individuals [27]. The evidence suggests an epidemiologic synergy between both diseases, and HSV-2 has been postulated as the most important STI driving the HIV prevalence in sub-Saharan Africa [43].

We included malaria as an example of a parasitic infection, given its geographical overlap with HIV and its high prevalence in Malawi. Malaria is endemic in all parts of Malawi and many other countries in sub-Saharan Africa. According to The World Health Organization, 6 million of episodes of malaria occurred in 2006, accounting for about 33% of all outpatient visits in Malawi. Additional file 1, Table S2 lists the key assumptions of the epidemiological module.

A key assumption for the epidemiological module is that the interaction caused by co-infection has only one direction. In other words, we assumed that HIV infection has no effect on the natural history of the other infectious diseases included in the model. This assumption may be seen as an oversimplification because studies have shown that HIV infection affects the transmission and progression of other infectious diseases such as HSV-2 and malaria. Yet, studies have mainly focused on the impact of co-infection on HIV. As a result, uncertainty about the effect of co-infection on the other diseases is still high.

The core of our model is the spread of HIV infection through penile-vaginal contact. Before the introduction of HIV infected individuals, the model simulates for several months the dynamic of the other infectious diseases previously mentioned. When an endemic steady state for all infectious diseases is reached (after about 500 monthly time steps), the model introduces HIV infected individuals until the HIV prevalence reaches 1%, which is the prevalence observed in Malawi in 1981 [60].

For our simulation, the algorithm assessed whether the individual infected with HIV has another infectious disease, and if co-infection was present, the HIV transmission probability was increased depending on the amplification factor. Then, the new HIV transmission probability including the amplificatory effect was calculated by

where *T *is the stage and sex-specific transmission probability per sexual contact (Additional file 1, Table S2). The HIV transmission probability per partnership per month is then calculated using the binomial (Bernoulli) model as

where *C_n _*is the number of sexual contacts the individual has with the partner.

Cofactor values of the STI's included in the model were obtained from population-based estimations expressed as odds ratios and relative risk per sexual contact. For malaria, we assume that the enhancement on the transmission probability per sexual contact depends on the logarithmic (base 10) incremental change in the viral load according to . The 2.45 factor is the rate ratio increase in transmission probability with each one-log increment in viral load [27], and log_10 _(*vl*) is the logarithmic (base 10) increment of the plasma viral load; see [45] for malaria increment data. Cofactor values included in the model are listed in Additional file 1, Table S2.

When multiple co-infections are present, we assumed a saturation effect of the enhancement on the transmission probability. Thus, when more than one co-infection is present, the transmission probability is amplified only by the highest cofactor. For the special case of HSV-2, the amplification factor is only effective if the HSV-2 infection is reactivated (shedding) [37,61]. Therefore, the algorithm not only verifies the presence of HSV-2 co-infection but also its reactivation. On the other hand, HSV-2 not only enhances the transmissibility of HIV but also affects the susceptibility to being infected with HIV [61]. For this reason, the algorithm verifies if the susceptible receptor is infected with HSV-2 and its reactivation stage. In this case, the transmission probability is also increased by the respective amplification factor. A detailed description of the methodology can be found in the Additional file 1, Text S1.

### Calculation of the epidemiologic synergy

HIV infections caused by co-infection with other infectious diseases may also generate secondary HIV infections, regardless of the presence of co-infection [43]. Therefore, the HIV prevalence measures both the HIV transmissions caused by the direct biological effect of co-infection and the secondary or indirect infections caused by co-infection. We estimated the effect of co-infection on the dynamic of the HIV epidemic in the sexual network by comparing the prevalence for different scenarios: no co-infection, all co-infections (default scenario), no HSV-2 co-infection, and no malaria co-infection. We also measured the direct effect of co-infection on the HIV incidence by using population attributable fractions (PAF) [62-64]. We estimated the PAF of HIV incidence attributable to all co-infections, HIV-2, malaria, and gonorrhea and syphilis. The PAF is calculated by

where *IR _nocofactor _*(*t*) is the incidence rate of HIV in the different scenarios with the cofactor effect removed at time *t*, and *IR _defaultcofactor _*(*t*) is the HIV incidence rate with the default cofactor effect at time *t *[43,65].

To identify the epidemiologic synergy at different periods of the HIV epidemic, we calculated the PAF for different time points in a separate set of simulations by removing the cofactor effect on HIV transmission over two years, starting at times (*t*) 0, 8, 15, and 20 after the introduction of HIV. This allows us to measure of the direct role of co-infection in HIV incidence at each time point (*t*). Results from all different scenarios are based on means over 200 simulations.

### Uncertainty and sensitivity analyses of the key parameters

To conduct uncertainty and sensitivity analyses of the key parameters, we adopted the Latin Hypercube Sampling/Partial Rank Correlation Coefficient (LHS/PRCC) technique [66,67]. In LHS, the estimation of uncertainty for each key parameter is modeled by treating each input parameter as a random variable with a uniform probability distribution function. Upper and lower bounds on these distributions were assigned based upon the available data.

To study the uncertainty of the parameters for the two different modules, we conducted three different uncertainty analyses. For the behavioral module, the simulations did not include the amplification cofactor caused by co-infection. Hence, we used the default probability of HIV transmission *T = *0.003 and we performed a LHS of the more important behavioral parameters. For the epidemiological module, the simulations included the amplification co-factor caused by confection and we conducted a LHS of the cofactor values. In the third uncertainty analysis, we performed a LHS of both the behavioral parameters and the cofactor values. 200 simulations were run for each uncertainty analysis. The variability in the outcome variable (HIV prevalence) was then estimated by simple descriptive statistics. Sensitivity analyses were then performed by calculating PRCCs for each input parameter. The details of these analyses can be found in the Additional file 1, Text S2.

## Results

### Sexual network

The graphical representation of the degree distribution (i.e. number of sexual partners per year) for both males and females shows that the gamma distribution provides a good fit to the data (Additional file 1, Figure S1). For males, the mean number of sexual partners per year was *μ *= 2.12, with standard deviation SD = 1.23, and with a scale parameter *θ *= 1.1 and shape parameter *k *= 1.9 for the gamma distribution. For females, the mean number of sexual partners per year was *μ *= 1.84 with standard deviation SD = 1.04, and with scale parameter *θ *= 0.4 and shape parameter *k *= 3.8 (Additional file 1, Table S1).

The resulting number of sexual partners per year obtained from the simulations for both males and females seems to agree with the estimated annual degree distribution from Malawi (Additional file 1, Figure S1), the age distribution of the entire population (Additional file 1, Figure S2), and the age distribution of married individuals (Additional file 1, Figure S3). We found, however, that individuals accumulated more long-term relationships (marriages) throughout their entire sexual life than indicated by the data (Additional file 1, Figure S4). This inconsistency could result from a low estimate of the average duration of marriage (~7 years) in the simulations.

Since long-term relationships increase the number of exposures, the risk of transmission or acquisition of the virus also increases. Consequently, the observed discrepancy between the lifetime number of long-term relationships observed in the simulations and the data may generate an overestimation of the HIV prevalence.

Using the number of connections per month (see Additional file 1, eq. 9) and the number of connections generated by individuals who already have one connection, we found that the frequency of concurrency estimated from the model was 0.3. In other words, 30% of the individuals who had a sexual partner were in at least one concurrent relationship. This value seems to be consistent with some estimates of concurrency from sub-Saharan Africa [68]. See also [69], where the concurrency estimate was higher, but concurrent relationships were not clearly distinguished from serial monogamy [70].

### Simulation 1: no co-infection

**Figure 1:**
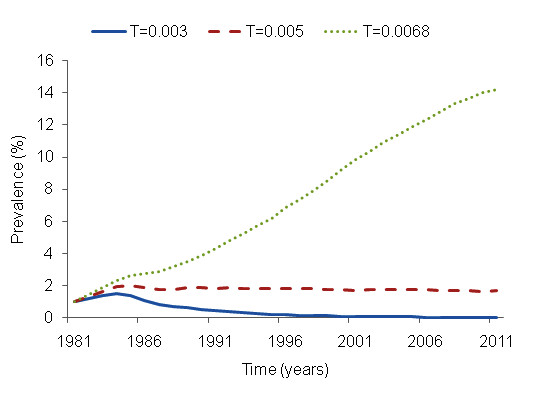
**HIV epidemic curves with constant probability of HIV transmission (no co-infection)**. At *T *= 0.003 no epidemic can be generated, and HIV prevalence decreases leading to extinction (prevalence estimated for 2010 = 0%)). With a *T *= 0.005 the infection can persist and reached and endemic steady state at a prevalence equal to the initial prevalence (HIV prevalence estimated for 2010 = 1%). At *T *= 0.0068 the epidemic curve generated is similar to the HIV epidemic observed in Malawi (HIV estimated prevalence for 2010 = 10%).

The first simulation, which included the most recent estimate of the male-to-female probability of HIV transmission *T *= 0.003 [20], suggests that with this constant probability of HIV transmission the infection does not survive in the population: no epidemic can be generated, and HIV prevalence decreases to extinction (Figure 1). On the other hand, with *T *= 0.005 we observed that the infection can persist and reached an endemic steady state at a prevalence equal to the initial prevalence (1%). Finally, with a *T *= 0.0068 we could generate an epidemic curve similar to the HIV epidemic observed in Malawi (Figure 1). This value is slightly above the 95% confidence interval estimated for male-to-female probability of HIV transmission [20] (see details in Additional file 1, Text S2).

### Simulation 2: amplification cofactor

**Figure 2:**
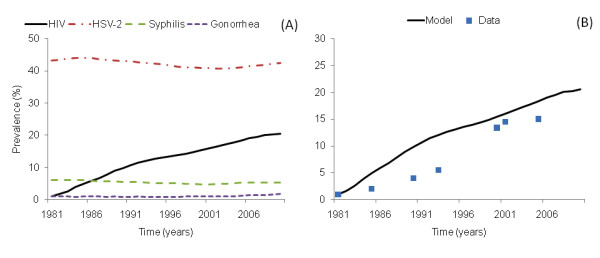
**Time course of HIV with the inclusion of the amplification effect caused by co-infection (simulation 2)**. In (A), the dynamics of HSV-2, syphilis, gonorrhea and malaria were simulated and allowed to approach steady state before the introduction of HIV into the population. Once endemic steady states of these infections were reached, 1% of individuals were infected with HIV, and HIV established in the population. (B) shows in more detail the time course of HIV in Malawi simulated by the inclusion of the amplification effect generated by co-infection (Simulation 2). The measured HIV prevalence from Malawi, for both males and females in general population, was extracted from several studies [91].

For this simulation, we used the baseline probability of HIV transmission for low income countries *T *= 0.003 [20], but we also included the amplification cofactor for HIV transmission generated by co-infection with HSV-2, gonorrhea, syphilis and malaria. The results from this scenario showed that the amplification effect caused by co-infection was sufficient to generate an HIV epidemic curve similar to the curve observed in Malawi (Figure 2B, HIV prevalence estimated for 2005 ~17%).

From our model, we observed that the prevalence for gonorrhea (~2%), syphilis (~4%), HSV-2 (~40%) and malaria prevalence in dry (30%) and rainy (40%) seasons resemble the prevalence observed for these STIs in Malawi and other countries of sub-Saharan Africa (Figure 2A) [60,71-77].

Even when parameter values of the model were obtained independently of the Malawi data, our results resemble the HIV prevalence as well as the age distribution prevalence observed in that country. As reported in the data, the female HIV prevalence was higher than the male prevalence; the resulting age distribution of prevalence, however, differs somewhat from the distribution reported for Malawi. In our results, the peak of the prevalence is located at ages 25-29 for both males and females, while the data suggest that the peak is at ages 30-34. The model also overestimates the HIV prevalence at early ages (Additional file 1, Figure S5 A, B).

We compared the simulated age distribution of HSV-2 prevalence with the observed prevalence in Malawi. The age distribution for HSV-2 obtained from the simulation is consistent with the data, but the resulting HSV-2 prevalence is somewhat lower than the prevalence observed in Malawi (Additional file 1, Figure S5 C, D).

**Figure 3:**
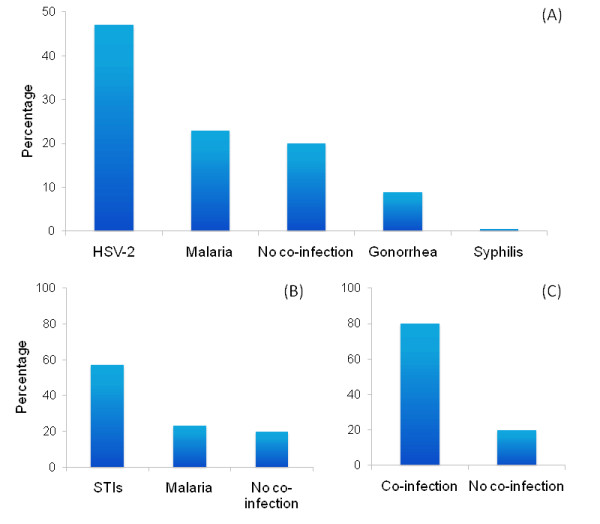
**Fraction of new infections when the HIV infectious (transmitter) individual has a co-infection (simulation 2)**. The model tracks the mechanism of the transmission of the infection. For the simulated period, if an HIV infection takes place, we recorded the presence in the infectious individual of any of the four infectious diseases included in the model, or in the case of HSV-2 due to the enhanced susceptibility caused by HSV-2 infection, we also recorded the presence of this infection on the susceptible individual and identified the cause of the amplification of the transmission caused by co-infection. (A) shows that HSV-2 was the most prevalent infection in co-infected individuals who transmitted the HIV infection, followed by malaria. This result is associated with the high prevalence of both infections in the general population. Grouping all STIs, more than 50% of the HIV infections were associated with an STI (B). More than 70% of the total HIV infections were caused by co-infected individuals (C).

Our results also showed that more than 40% of the HIV infections were associated with HSV-2 co-infection (Figure 3A), and more than 70% of the total HIV transmissions were associated with STI or malaria co-infection (Figure 3C). Significantly, these values indicate that transmission was commonly by co-infected individuals, but the results do not estimate the incidence of HIV due to the direct effect of co-infection.

### Simulation 3: No HIV-HSV-2 co-infection

**Figure 4:**
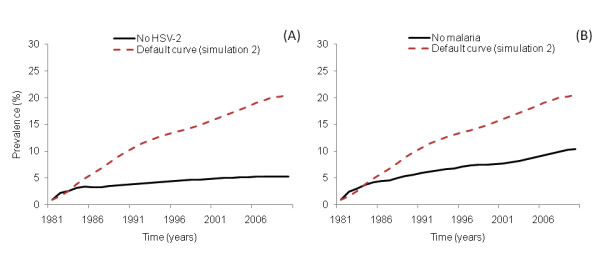
**Removing the amplification effect on HIV transmission**. (A) shows the time course of HIV when HSV-2 does not have an amplification effect on HIV (simulation 3) and the prevalence for 2005 decays to ~6% (C). In the scenario where malaria does not have an amplification effect on HIV transmission (simulation 5), the resulting prevalence decays to ~10% (B).

Because HSV-2 was the most important infectious disease in terms of co-infected individuals who transmitted the HIV infection (Figure 3A), we simulated the absence of the amplificatory effect generated by HSV-2. For this simulation, the prevalence decreased to ~6% in comparison with the prevalence observed in simulation 2 (Figure 4A).

### Simulation 4: No HIV-malaria co-infection

Without the malaria amplification effect, the HIV prevalence declined to ~10%, indicating that malaria contributed ~7% to the overall HIV prevalence (Figure 4B). This result is higher than the result obtained by Abu-Raddad and coworkers [78], who simulated the co-infection effect of malaria on HIV infection in Kisumu, Kenya, using a system of differential equations. They found that excess of HIV prevalence caused by malaria was 2.1%.

### Population Attributable Fraction (PAF)

**Figure 5:**
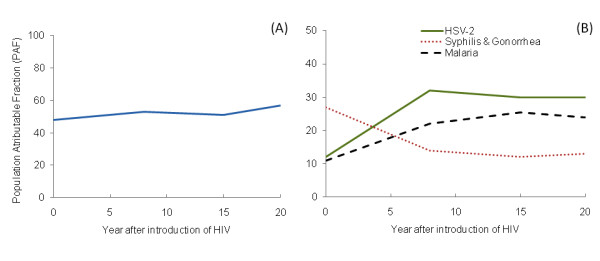
**Population Attributable Fraction (PAF) due to co-infection**. We measured the direct effect of co-infection on the HIV incidence by using the population attributable fractions. (A) The PAF due to co-infection in general, and (B) the PAF due to HSV-2 (solid line), syphilis and gonorrhea (dotted line) and malaria (dashed line).

The results indicated that the proportion of new HIV infections due to all co-infections together was > 50% and that this value remained constant over time (Figure 5A). When we obtained the PAF for each infection, however, we observed different patterns throughout the simulation. At the beginning of the HIV epidemic, infections such as gonorrhea and syphilis had the highest PAF (~26%). Over time, their influence on the spread of HIV decreased to a final PAF of 13%, 20 years after the introduction of HIV (Figure 5B). In contrast, common infections in the general population such as malaria and HSV-2 had a low initial PAF (~12%), followed by an increase. In agreement with previous studies [43,79] we found that the PAF due to HSV-2 increases during the first 10 years and then remained constant after 15 years from HIV introduction at the highest PAF (~30%).

Likewise, the proportion of new HIV infections caused by malaria increased during the early years of the epidemic, and then remained constant after 15 years from HIV introduction, with a PAF of ~ 25% (Figure 5B); this value is in close agreement with previous results for eastern sub-Saharan Africa based on regression analysis [80].

## Discussion

Our results indicate that a data-supported fixed value for HIV infectivity fails to describe the dynamics of the epidemic. Regardless of the low probability of heterosexual transmission per sexual contact, the inclusion of individual variation in HIV infectivity generated by transient but repeated increases in HIV viral loads associated with co-infections may substantially increase the transmission rate [25].

Our model thus suggests that the HIV epidemic in sub-Saharan Africa may be explained by heterosexual transmission, and supports the hypothesis that variation among individuals and through time caused by biological cofactors such as co-infection may have triggered the vast HIV epidemic observed in sub-Sahara Africa. The high prevalence of infectious diseases such as malaria and HSV-2 probably provided suitable conditions for the spread of the infection in the general population.

The remarkably high HIV prevalence observed in sub-Saharan Africa may thus reflect the particular environment at the early and mature stages of the epidemic that are unique to this part of the world [43]. These results highlight the possibility that co-infection is a necessary rather than merely a contributing factor in the successful spread and survival of HIV in populations where heterosexual vaginal-penile contact is the main mechanism of transmission.

According to our results, 50% of all new HIV infections throughout the epidemic can be attributed to co-infection with the infectious diseases included in the model. However, we observed opposite time trends in the contribution from two infections with low prevalence in the general population (i.e. decreasing trends for gonorrhea and syphilis), and from infections with high prevalence (i.e. increasing trends for HSV-2 and malaria).

Some similar results have been obtained in previous studies [43,65,79], but none has documented the pattern in PAF that we observed for malaria throughout the epidemic. Our model is the first to include not only the co-factor effect of other STIs on HIV transmission but also the co-factor effect of a parasitic disease in the same simulation. The expected high frequency of malaria-HIV co-infected individuals who transmitted the HIV infection (Figure 3A) raises the possibility that parasitic diseases like malaria with high prevalence in Africa, may be playing a similar role to that of an STI like HSV-2 in terms of new HIV infections. This similarity should be greatest in populations with a mature HIV epidemic and where both infections overlap geographically. Despite the low co-factor effect on HIV transmission generated by malaria, the high prevalence of this infection may have increased its effect on the HIV incidence as the HIV epidemic has invaded the general population.

Our analysis suggest that the synergy among sexually transmitted infections and parasitic infections allowed the HIV epidemic to reach the general population, which may not have been possible without the cofactor effect on HIV transmission generated by co-infection [43]. This in turn suggests that an HIV epidemic may be mitigated or halted through measures that decrease viral infectivity. The control and treatment of several common infectious diseases could decrease the incidence of HIV over the long-term.

Although interventions aimed at reducing the incidence of STIs have a prominent place in control strategies, some studies have failed to show an impact of STI treatments on HIV incidence [81,82]. Some authors have suggested that population differences in sexual behavior, differences in STI prevalence and the stage of the HIV epidemic may explain the poor impact of this control intervention [83]. These studies [81,82] commonly focused on STIs with low prevalence in the general population, such as syphilis, trichomoniasis, gonorrhea and chlamydia.

However, as our study and other studies [40,84,85] have indicated, infectious diseases present in the general population such as HSV-2 and parasitic diseases have the highest impact on the HIV incidence in mature HIV epidemics. A transmission study conducted to determine whether HSV-2 suppression in HIV/HSV-2 co-infected individuals reduces the risk of HIV transmission indicated that, despite a notorious reduction in the prevalence of genital ulcer diseases generated by HSV-2, and a 0.25 log_10 _copies/ml reduction in plasma HIV-RNA levels, HSV-2 suppression with acyclovir did not prevent HIV transmission [86]. In a subsequent work, however, Lingappa et al. demonstrated that a 0.74 log_10 _copies/ml reduction in HIV plasma RNA concentration is necessary to reduce the HIV transmission rate by half [87]. HSV-2 suppression with acyclovir may thus have been insufficient to yield a detectable reduction in HIV transmission risk. Lingappa et al. concluded that treatment of co-infections capable of reducing plasma HIV levels by > 0.7 log_10 _copies/ml may be a valuable tool for suppressing the transmission of HIV.

### Limitations of the model

The results of this study derive from a simulation model and depend on the validity of the underlying assumptions and parameter magnitudes. Uncertainties about the magnitudes of these parameters suggest that the conclusions presented here should be interpreted with caution. HIV transmission probability and the effect of behavioral and biological cofactors on HIV transmission require more thorough quantification [43,65,79], and we hope that our results will help motivate this work.

Additionally, cofactor values are commonly estimated from population-based observations of co-infection status in individuals or couples [88]. In these cases, the association between the transmission of HIV and the presence of an STI is generally expressed in terms of odd ratios, hazard ratios or relative risk per sexual contact [89]. These estimates, however, can be particularly difficult to interpret as a consequence of multiple potential biases.

To reduce confounding effects resulting from other behavioral and biological risk factors, estimates of cofactor effects are statistically adjusted for the influence of these risk factors. But these analyses may not completely control for the confounding effects because STIs, HIV and other behavioral and biological risk factors may cluster not only in study subjects but also in the unknown partners of the individuals included in the study [88]. Moreover, the confounding generated by the characteristics of the sexual network such as concurrency, mixing patterns and numbers of sexual partners is virtually impossible to control for completely [88].

On the other hand, the high variation in HIV prevalence produced by the model observed in the uncertainty analysis compromises the accuracy of the model's predictions (Additional file 1, Table S9). The sensitivity analysis indicated that the model is highly sensitive to the behavioral parameters that influenced the per-partner probability of HIV transmission, such as the average duration of casual relationships and the mean number of sexual contacts, and to the biological parameters responsible for the cofactor effect of infections present in the general population, such as malaria and HSV-2 (Additional file 1, Table S10). Thus, efforts focused on more precise estimations of these parameters will improve the accuracy of predictions from models exploring the causes of the HIV epidemic in sub-Saharan Africa.

Lastly, the model assumes that HIV is transmitted exclusively by penile-vaginal heterosexual contact, and thus the model does not include anal intercourse as a mechanism of HIV transmission. Since anal intercourse increases the probability of transmission [20], this type of sexual behavior has been proposed as an important risk factor of HIV transmission. Although many authors claim that heterosexual penile-vaginal contact is the main mechanism of transmission in sub-Saharan Africa, more of information about the frequency of anal intercourse, including men who have sex with men, would allow an evaluation of this possibly important but little-studied mechanism of transmission in the epidemic in sub-Saharan Africa [90].

## Conclusions

The present study shows that the natural history of HIV in sub-Saharan Africa cannot be fully understood if individual variation in infectiousness is neglected. Our model suggests that a single value for HIV infectivity fails to describe the dynamics of the epidemic. Regardless of the low probability of heterosexual transmission per sexual contact, the inclusion of temporal and individual variation generated by transient increases in HIV viral loads associated with co-infections may provide a biological basis for the accelerated spread of HIV in sub-Saharan Africa.

Our findings have direct implications on the design of mathematical models attempting to replicate the epidemic curve observed in sub-Saharan Africa, as well as control interventions focusing on decreasing the incidence of HIV. If highly infectious individuals can be identified, then the efficiency of control measures could be greatly increased. The realization of these kinds of targeted interventions, however, requires a better understanding of factors determining individual infectiousness such as co-infections.

## Competing interests

The authors declare that they have no competing interests.

## Authors' contributions

DFC wrote the draft manuscript and collaborated on project conception, model design, and simulation programming. PHC collaborated on project conception, model design and helped in writing the manuscript. BA collaborated on project conception, model design, and simulation programming. SLS collaborated on project conception and model design. GGR collaborated on project conception and helped in writing the manuscript. All authors read and approved the final manuscript.

## Pre-publication history

The pre-publication history for this paper can be accessed here:

http://www.biomedcentral.com/1471-2334/11/216/prepub

## Supplementary Material

Additional file 1 **Supplementary material**. A single PDF file. 31 pages that include supplementary methods, and supplementary results. The supplementary tables and figures are embedded in the file. Click here for file
