# Supplementary material for: Effect of variable transmission rate on the dynamics of HIV in sub-Saharan Africa
Source: BMC Infect Dis. 2011 Aug 11;11:216. doi: 10.1186/1471-2334-11-216 (PMC3175213; doi:10.1186/1471-2334-11-216)
Supplement: Additional file 1 — Supplementary material. A single PDF file. 31 pages that include supplementary methods, and supplementary results. The supplementary tables and figures are embedded in the file. [file 1471-2334-11-216-S1.PDF]

Effect of variable transmission rate on the dynamics of HIV in sub-Saharan Africa

Diego F Cuadros, Philip H Crowley, Ben Augustine, Sarah L Stewart, Gisela García-Ramos

Additional file 1 - Supplementary material

Text S1: Supplementary methods
Behavioral module.......... 2
Epidemiological module.......... 6
Text S2: Supplementary results
Uncertainty and sensitivity analyses of the behavioral module.......... 15
Uncertainty and sensitivity analyses of the epidemiological module.......... 18
Uncertainty and sensitivity analyses of the complete model.......... 19
Limitations of the model.......... 21
Supplementary figures.......... 23

Text S1: Supplementary methods

Sexual partnerships were assumed to be exclusively heterosexual, and two types of partnerships, distinguished by duration, were considered. The population was assumed closed and stable, with only maturation and mortality into and out of the sexual network. The population size remained constant, with individuals maturing into the network to offset those who die or mature out of the network. In accord with the highest resolution of relevant data, a monthly time step was used. With this model, the effects of network structure on disease transmission, relationship type, and co-infection with other infectious diseases were evaluated.

Behavioral module

Building Individuals

In the model, equal numbers of individuals of each sex were created and assigned an age and node degree (maximum number of partners per year). The initial age was assigned with a gamma probability distribution, with a range between 15 to 60 years old (Table S1, parameters 5-8).

Consequently, individual age was used to determine when individuals should be removed from the sexual network, and was the basis for other age-specific traits.

We adopted a likelihood framework to estimate model parameters of the annual degree node distribution by using the number of sexual partners reported in the Malawi study for both males and females. For both males and females, the reported number of sexual partners follows a gamma distribution:

$$f(n) = n^{k-1} \frac{e^{-n/\theta}}{\Gamma(k)\theta^k}, \quad (1)$$

where the annual degree distribution is defined as the frequency of node degrees $\boldsymbol{n}$ in the network, with shape parameter k and scale parameter θ.

Because the time step of the simulation is one month, and with the aim of preserving the degree distribution of the network, we estimated the monthly probability of generating a partnership according to the degree node for each individual, and we calculated the maximum number of connections that should be generated in the network each month. We assumed that casual partnerships were temporally independent, began at uniformly distributed random times throughout the year, and lasted for a set duration, d. Further, as marriages generally last more than one year, we assumed that if an individual was married, the relationship did not end during any observation year.

Thus, the expected number of casual partnerships within a year, c_y, is the expected number of marital partnerships, m_y, subtracted from the expected number of total partnerships, n_y or

$$c_y = n_y - m_y . \quad (2)$$

P_m, the probability that a casual partnership recorded within the year, would also be recorded within one particular month of the year given by

$$p_m = \frac{1+d}{12+d} . \quad (3)$$

Here a partnership is recorded within the observation month if its center point falls within an interval of length 1+*d* centered on the middle of the month, and a partnership is recorded within the observation year if its center point falls within an interval of length 12+*d.* Because yearly casual partnerships are assumed to be temporally independent, the expected number of monthly partnerships, n_m, for an individual with n_y yearly partnerships is the expected number of yearly casual partnerships, plus the expected number of yearly marital partnerships, m_y.

$$n_m = c_y p_m + m_y = \frac{(n_y - m_y)(1+d)}{12+d} + m_y . \quad (4)$$

The vector n_m was used for estimating the probability of generating a connection according to the node degree of each individual.

For calculating the maximum number of connections that should be generated each month, we assumed that the expected numbers of yearly casual partnerships are Poisson distributed; therefore, for each n_m, the probabilities that the expected c_y partnerships generate n_m casual partnerships during the month, are found according to

$$p(c_y, n_m) = \frac{c_y^{\,n_m} e^{-c_y}}{n_m!} , \quad (5)$$

evaluated from n_m = 0 to n_{max}. These probabilities are then weighted by the yearly degree frequencies, correcting for the fraction of individuals that are married, and summing over

partnership numbers. The probabilities for casual partnerships, weighted by the node degree distribution $f_y(n)$ are

$$p_c(c_y, n_m) = p(c_y, n_m) * (1-m) * f_y(n_m) \,. \quad (6)$$

The probabilities for marital partnerships, weighted by $f_y(n)$ are

$$p_m(c_y, n_m) = p(c_y, n_m) * m * f_y(n_m) \,. \quad (7)$$

The probabilities for all partnerships, weighted by $f_y(n)$ are

$$p_t(c_y, n_m) = p_c(c_y, n_m) + p_m(c_y, n_m) \,. \quad (8)$$

The total monthly partnership frequencies are then

$$f_t(n_m) = \sum_{c_y=1}^{c_{\max}} p_t(c_y, n_m) \,. \quad (9)$$

Each frequency includes both males and females; therefore, we divided it by two to obtain the maximum number of connections that should be generated in one month.

Forming Initial Partnerships

We allowed for two types of relationships; casual relationships, which lasted on average 6 months (Table S1 parameter 14), and marriage (long term relationship), which lasted more than 1 year. Partnership formation occurs in two steps. First, the age mixing pattern of marriage is generated by estimating the probability of generating a marriage using a gamma probability distribution (eq 1) according to age and gender (Table S1, parameter 9-12). This step generates the age mixing pattern of marriage. Then marriages are generated until the fraction of the married population estimated for Malawi is reached (Table S1, parameter 13). Casual partnerships are then formed with the remaining available individuals (married or single) according to their node degree, and then these available individuals are randomly connected until the monthly number of connections f_y (eq 9) is reached.

Dynamic of the Network

Partnerships are broken and reformed with data-determined probabilities each month; thus, with the aim of keeping the yearly degree distribution, individuals may sometimes have zero partnerships in a particular month. For Malawi, the probability of ending a marital relationship follows an exponential distribution as a function of the duration of the marriage X, with an average duration of marriage of 6.92 years. Hence, each marriage has a probability of ending in each time step according to an exponential cumulative probability function with a scale parameter λ (eq 10). Casual partnerships break up following the same function but at a higher rate λ (Table S1, parameter 14):

$$f(x) = 1 - e^{-\lambda x}. \quad (10)$$

As the sexual network represents only individuals engaging in sexual activity, population dynamics consist of maturation into and out of the sexual network by “mortality” related to age or HIV. At a maximum age of 60, individuals are assumed to no longer be sexually active and are removed from the sexual network and replaced with a new individual of age 15. This new individual has the same sex and node degree as the removed individual, so that the sex ratio and network structure is maintained. Individuals who have broken a relationship and new individuals who have replaced deceased individuals or those who have matured out of the sexual network are reconnected by the same process used to make the initial connections.

Table S1- Parameters used in the behavioral module of the model

Parameter	Value	Reference
Number of sexual partners per year		
Gamma distribution (males)		Derived from [1]
1. scale	1.1	
2. shape	1.9	
Gamma distribution (females)		Derived from [1]
3. scale	3.8	
4. shape	0.4	
Initial condition age distribution		
Gamma distribution (males)		Derived from [2]
5. scale	2.24	
6. shape	17.32	
Gamma distribution (females)		Derived from [2]
7. scale	2.46	
8. shape	13.5	
Marriage age distribution		
Gamma distribution (males)		Derived from [1]
9. scale	2.25	
10. shape	17.23	
Gamma distribution (females)		Derived from [1]
11. scale	2.38	
12. shape	13.85	
13. Percentage of married population	63%	Derived from [1]
14. Duration of casual relationship	6 months	Derived from [1]
Separation probability (exponential distribution)		
15. scale	0.15	Derived from [1]

Epidemiological module

The epidemiological module was subdivided into two steps, the spread of the infections, and the progression and recovery of each infection. We assumed that HIV transmission is caused by penile-vaginal heterosexual contact exclusively. We selected gonorrhea, syphilis and herpes simplex virus type-2 (HSV-2) as the infections to be simulated in the sexual network, based on the amplification effect on HIV transmission and their relevance in terms of prevalence in the

Malawi population. The dynamics of these STI's are well known, and the effect of each infection on the transmission of HIV has been determined.

A key assumption for the epidemiological module is that the interaction caused by co-infection has only one direction. In other words, we assumed that HIV infection has no effect on the natural history of the other infectious diseases included in the model. This assumption may be seen as an oversimplification due to the fact that studies have shown that HIV infection affects the transmission and progression of other infectious diseases such as HSV-2 and malaria. Yet, studies have mainly focused on the impact of co-infection on HIV. As a result, uncertainty about the effect of co-infection in the direction of the other diseases is still high.

Spread of the Infections

For the spread of the STI's, the probability of disease transmission per partnership per month depends on the number of sexual contacts per partnership per month and the transmission probability per sexual contact for each STI. The number of sexual contacts per partnership per month is assumed to be a function of the number of partnerships of the infected sex only. This function is defined as:

$$C_n = C_1 N^{-0.75} \quad , \quad (11)$$

where C_1 is the number of sexual contacts per month for individuals having one partnership according to age (Table S2 parameter 7-10) , and N is the number of partnerships the individual has in the specific month. This function is consistent with individuals with more partnerships having more sex but keeps the number of sexual contacts per month realistic.

For each infection, we obtained the estimated amplification factor on the probability of HIV transmission per sexual contact. For our simulation, the algorithm assessed whether the individual infected with HIV has another infectious disease, and if co-infection was present, the HIV transmission probability was increased depending on the amplification cofactor (Table S2

parameter 49-53). If co-infection is not present, then the cofactor value is equal to one. Then, the new HIV transmission probability including the amplificatory effect was calculated by

$$T_c = T * cofactor \,, \quad (12)$$

where ***T*** is the stage or sex-specific transmission probability per sexual contact (Table S2 parameter 1-6). When multiple co-infections are present, we assumed a saturation effect on the enhancement on the transmission probability. Thus, when more than one co-infection is present, the transmission probability is amplified only by the highest cofactor. For the special case of HSV-2, the amplification factor is only effective if the HSV-2 infection is reactivated (shedding) [3]. Therefore, the algorithm not only verifies the presence of HSV-2 co-infection but also its reactivation. On the other hand, HSV-2 not only enhances the transmissibility of HIV but also affects the susceptibility to being infected with HIV [3]. For this reason, the algorithm verifies if the susceptible receptor is infected with HSV-2 and its reactivated stage. In this case, the transmission probability is also increased by the respective amplification factor (Table S1 parameter 53).

The transmission probability per partnership per month is then calculated using the binomial (Bernoulli) model as

$$T_p = 1-(1-T_c)^{c_n} \,, \quad (13)$$

where C_n is the number of sexual contacts the individual has with the partner (eq 11). The probability of condom use is represented by a gamma probability function (eq 1), depending on age (Table S2 parameter 31, 32). If a condom is used, then the probability of transmission of the STI is reduced 94% (Table S2, parameter 33):

$$T_{condom} = T_p * 0.06 \,. \quad (14)$$

Circumcision is known to affect the dynamics of HIV and has been proposed as one of the explanatory variables for contrasting differences in prevalence among countries of sub-Sahara Africa. The probability of HIV transmission from infected female to uninfected male is approximately halved by circumcision,

$$T_{circum} = T_p / 2 \ . \quad (15)$$

Circumcision is included in our model at the beginning of the simulation, where a percentage of males estimated for Malawi (Table S2 parameter 62) are randomly selected from the male population and identified as circumcised males.

For the spread of Malaria infection, we used the daily Entomological Inoculation Rate (EIR), defined as the number of infected bites that a person receives per day, for both rainy and dry seasons estimated for Malawi, as well as the malaria transmission efficiency for an infected bite. Each individual was exposed to a malaria infection by a probability of transmission:

$$T_{malaria} = \text{EIR} * \text{malaria transmission efficiency.} \quad (16)$$

Because $T_{malaria}$ is calculated based on daily EIR, while the time step of the simulation is the month, we calculated the monthly probability of malaria infection, $TM_{malaria}$, using the Bernoulli model:

$$TM_{malaria} = 1 - (1 - T_{malaria})^{30} \ . \quad (17)$$

In order to include the effect of partial immunity generated by multiple malaria infections, each new infection is recorded and then used for calculating the susceptibility of a new infection using the model proposed by Gu and coworkers [4]:

$$S_{malaria} = \frac{1}{1 + \frac{N_{\inf}{}^{\sigma}}{N_1}} \ , \quad (18)$$

where N_{inf} is the number of malaria infections an individual has had in his/her life; σ and N_1 are constants. After calculating the susceptibility coefficient, the new probability of malaria infection $TM'_{malaria}$ becomes

$$TM'_{malaria} = TM_{malaria} * S_{malaria} \ . \quad (19)$$

The core of our model is the spread of HIV infection. Before the introduction of HIV infected individuals, the model simulates for several years the dynamic of the other infectious diseases previously mentioned. When an endemic steady state for all infectious diseases is

reached (after about 500 time steps), the model introduces HIV infected individuals until the HIV prevalence reaches 1%, which is the prevalence observed in Malawi in 1981 [5].

HIV transmissions caused by commercial sex

The data available show that males do not report sexual contacts with prostitutes as sexual partners; as a result, the degree node distribution estimated for males does not take into account sexual contacts with prostitutes [6-8]. However, because commercial sex has been considered an important risk factor for acquiring HIV infection, we include this mechanism of HIV transmission in the model.

Data from another study in Cameroon, Kenya and Zambia, called the four cities study, was used to parameterize the spread of HIV infections caused by sexual contacts with prostitutes [7-8]. In our model, commercial sex is a simple generalized linear equation for calculating the probability that a male gets any of the STI's included in our simulation, including HIV. We assumed that in average 8% of the male population have six sexual contacts with a prostitute per year. The model is a mass action Bernoulli model, where the probability of transmission depends on the actual prevalence of the STI into the population. For HIV, because prostitutes are cataloged as a core group, and the prevalence is usually higher compared to the total population, we assumed that the HIV prevalence in prostitutes is five times the actual HIV prevalence in the population. Thus, the probability of transmission caused by sexual contact with a female sexual worker (FSW) is calculated as;

$$I_{fsw}(t) = 1 - \{(1 - p_{fsw}(t)) + [p_{fsw} * (1 - T)^n]\}, \quad (20)$$

where $p_{fsw}(t)$ is five times the HIV prevalence of the total population at time t, T is the probability of female to male transmission in chronic stage of HIV infection (Table S2 parameter 21) and n is the number of sexual contacts that a male individual has with the prostitute in a month (Table S2 parameter 60). Every month, a number of male individuals (Table S2 parameter 54) are chosen randomly and become infected via commercial sex with a probability I_{FSW}. Because our goal was to measure the impact of co-infection on the spread of HIV infection, we

allowed to prostitutes to have infections with other STIs according to the prevalence of the STI reported for prostitutes. Hence, the baseline HIV transmission probability was amplified according to the presence of co-infection.

Progression of the Infections

The model simulates the progression of each infection. Gonorrhea is the simplest one, where each month infected individuals have a constant probability of recovery from the infection, with an average duration of the infection of ten weeks (Table S2 parameter 30). An individual that recovers from gonorrhea infection becomes susceptible again.

Syphilis infection has a more complex progression divided in two main stages. The primary stage of the infection is the first six months, characterized by a high probability of transmission for both sexes and the highest amplificatory effect on HIV transmission (Table S2 parameter 34, 36). After this initial six month stage, the infection remains latent for eight years. The latent stage is characterized by a low probability of transmission and no amplification effect on HIV transmission (Table S2 parameter 35-37). We assume that individuals infected with syphilis receive treatment; therefore, after the latent stage, individuals recover from the infection and become susceptible again.

HSV-2 infection has the most complex dynamics. A newly infected individual enters a chronic stage of the infection lasting 10 years. During this interval, there are about four reactivations of the infection (shedding) per year for non HIV co-infected individuals, and six episodes per year for co-infected individuals (Table S2 parameter 25,26). These reactivation periods last 15 days and produce the amplificatory effect of HSV co-infection on both the transmission and acquisition of HIV (Table S2 parameter 27). Hence, for our simulation, only co-infected individuals with HSV-2 in shedding episodes have amplified the transmission and acquisition of HIV.

In contrast to the other STI's included in the simulation, individuals infected with HSV-2 do not recover from the infections; but after the 10-year chronic stage, individuals do not

transmit the HSV-2 infection, and there is no cofactor effect on HIV transmission or acquisition (Table S2 parameter 23, 24).

For malaria, infected individuals recover at a constant rate, with an average duration of the infection of two months ($R_{malaria}$). However, with the aim of including the effect of partial immunity generated by multiple infections, we used the equation proposed by Gu and coworkers [4], to calculate a recovery coefficient $r_{malaria}$, which decreases the duration of the infection:

$$r_{malaria} = \frac{1}{1 + \frac{N_{\inf}{}^{\rho}}{N_2}} . \quad (21)$$

Here N_{inf} is the cumulative lifetime number of the individual's malaria infections; ρ and N_2 are constants. After calculating the susceptibility coefficient, the new probability of recovery from malaria infections $R'_{malaria}$ becomes

$$R'_{malaria} = R_{malaria} * r_{malaria} . \quad (22)$$

In order to maintain the concept of partial immunity, the recovery coefficient was truncated at 20 malaria infections with a minimum value of 0.5.

Individuals infected with HIV have a probability of developing AIDS calculated by a Weibull cumulative probability function depending of the duration of the infection $\boldsymbol{x}$, with scale parameter $\boldsymbol{\lambda}$, and shape parameter $\boldsymbol{k}$ (Table S2, parameters 11,12):

$$f(x) = 1 - e^{-(x/\lambda)^k} . \quad (23)$$

We assumed that individuals who develop AIDS are no longer spreaders of the infection; they leave the sexual network and are replaced by new individuals in the same way that was explained previously for “mortality”.

Table S2 - Parameters used in the epidemiological module of the model

Parameter	Value	Reference
HIV probability of transmission per sexual contact T (low income countries)		[9]
1. Acute male-female	0.0276	
2. Chronic male-female	0.003	
3. Advanced male-female	0.0219	
4. Acute female-male	0.03496	
5. Chronic female-male	0.0038	
6. Advanced female-male	0.02774	
Number of sexual contacts per month (by age)		[10]
7. 15-20	11	
8. 21-30	9	
9. 31-40	9	
10. 41-60	7	
Initial HIV infection age distribution (gamma)		Derived from [2]
11. Scale	8	
12. Shape	5	
HIV progression (Weibull cumulative probability function)		[11-12]
13. Scale	5	
14. Shape	4	
HSV-2 probability of transmission per sexual contact		[13-14]
Primary infection		
15. Male to female	0.3	
16. Female to male	0.15	
Early stage		
17. Male to female	0.01	
18. Female to male	0.005	
Chronic stage		
19. Male to female	0.005	
20. Female to male	0.003	
HSV-2 stage duration		[13-14]
21. Primary infection	3 weeks	
22. Early stage	3 months	
23. Chronic stage	10 years	
24. Latent stage	lifetime	
HSV shedding frequency per year		[14]
25. In HIV positive individual	6 per year	
26. In HIV negative individual	4 per year	

27. Shedding during reactivation	15 days	
Gonorrhea probability of transmission		[15-16]
28. Male to female	0.23	
29. Female to male	0.13	
30. Gonorrhea duration of infection	3 months	[15-17]
Condom use by age (Gamma distribution)		Derived from [2]
31. Scale	14.1	
32. Shape	1.78	
33. Condom reduction of transmission	94%	[18-19]
Syphilis probability of transmission		[15-16, 20-21]
34. Early stage male to female	0.175	
35. Latent stage male to female	0.018	
36. Early stage female to male	0.088	
37. Latent stage female to male	0.009	
Syphilis stage duration		[15-16, 20-21]
38. Duration early stage	6 months	
39. Duration latent stage	8 years	
Malaria Entomological Inoculation Rate (EIR)		Derived from [22]
40. Rainy season	1.2	
41. Dry season	0.6	
42. Duration of rainy season	6 months	[23-24]
43. Malaria transmission efficiency	0.026	
Malaria susceptibility coefficients		
44. Sigma	2	[4]
45. N_1	200	
Malaria recovery coefficients		[4]
46. Rho	2	
47. N2	400	
48. Time of malaria parasite clearance	2 months	[25-26]
Amplificatory factor in HIV transmission		
49. Gonorrhea	3	[27-32]
50. Syphilis	2.5	[32-35]
51. Malaria	1.25	Derived from [26, 36-37]
52. HSV-2	3	[3, 13-14, 32, 38-40]
53. Enhanced susceptibility to HIV in HSV-2 infected individual	3	[3, 13-14, 32, 38-40]
Commercial sex parameters		
54. Proportion of male population having sex with prostitutes per month	0.0066 (8% in a year)	[7-8, 41]
55. Gonorrhea prevalence in prostitutes	23%	[7, 41]
56. Syphilis prevalence in prostitutes	16%	[7, 41]

57. HSV prevalence in prostitutes	90%	[7-8, 41]
58. Percentage of circumcised males	31%	Derived from [2]
59. HIV reduction in probability of transmission in circumcised males	2 times	[42-43]
60. Number of sexual contacts a male individual has with a prostitute in a month	6	Derived from [7]

Text S2: Supporting results

Parameters included in our model are estimated from available published biological and behavioral data. Due to the variability generated by the different data collections and analyses, all estimations are associated with some degree of uncertainty. Analyses were conducted using Latin Hypercube Sampling and Partial Rank Correlation Coefficient (LHS/PRCC) through Monte Carlo sampling [44-45] from specific ranges using the uniform probability distribution function (pdf) for 200 runs of the model.

In the absence of published studies about the distribution and the range of most of the parameters, ranges were chosen to be as large as possible and represent plausible values for these parameters given the empirical evidence. These choices represent the most influential parameters in our formalism as seen in multiple analyses of the model and there are not precise estimates for them.

We conducted three different analyses where we explored the uncertainty and sensitivity of each module, behavioral and epidemiological, separately, and then we explored the uncertainty and sensitivity of the entire model.

Uncertainty and sensitivity analyses of the behavioral module

For the uncertainty and sensitivity analyses of the key parameters of the behavioral module, we set the default probability of HIV transmission T= 0.003, but we did not include the

cofactor effect caused by co-infection. We sampled seven parameters: the minimum and maximum age of individuals included in the sexual network, the mean number of sexual contacts per month (assuming the same for all ages), the mean durations of casual relationships, the proportion of the married population, the proportion of the male population that has sex with prostitutes per month, and the number of sexual contacts that a male has with a prostitute in a month. The specific range for the sampling of each parameter is specified in table S3.

Table S3 - Parameters sampled and their range for the uncertainty analysis for the behavioral module

Parameter	Range of uniform pdf	Reference
Minimum age	[14-18]	Derived from [2]
Maximum age	[55-62]	Derived from [2]
Number of sexual contacts per month	[6-14]	Representative assumption
Duration of casual relationship (months)	[3-9]	Representative assumption
Proportion of married population	[0.5-0.7]	Derived from [2]
Proportion of male population having sex with prostitutes per month	[0.005-0.01]	Representative assumption
Number of sexual contacts a male individual has with a prostitute in a month	[1-20]	Representative assumption

Descriptive statistics for the uncertainty analysis indicated that the variation generated by the uncertainty of the parameters sampled is fairly low (Table S4). On average, all possible combinations of the parameters sampled did not produce an epidemic. The 95th percentile, however, indicated that with some combination of the behavioral parameters and without the inclusion of co-infection, 5% of the simulations generated a HIV close to 5% (Table S4).

Table S4 - Descriptive statistics from the uncertainty analysis for the behavioral module in the absence of co-infections

Descriptive Statistics	HIV prevalence
Mean	0.87
Median	0
Standard Deviation	2.07
25th percentile	0
95th percentile	4.95

The sensitivity analysis revealed that the average number of sexual contacts per month and the mean duration of casual relationships were the only statistical significant parameters; in other words, these parameters may explain most of the variation observed among simulations (Table S5). The number of sexual contacts and the duration of the relationships are parameters related to the HIV transmission risk per partnership. According to the binomial model for the calculation of the per-partner probability of HIV transmission, increasing the duration of the relationship or the number of sexual contacts will increase the risk of HIV transmission. We observed that simulations with mean duration of casual relationship longer than 8 months and with 12 sexual contacts per month were able to produce an HIV epidemic higher than 6%.

Table S5 - Partial rank correlation coefficients of the parameters sampled from the behavioral module

Parameter	PRCC
Minimum age	0.02
Maximum age	0.07
Number of sexual contacts per month	0.49***
Duration of casual relationship	0.53***
Proportion of married population	0.04
Proportion of male population having sex with prostitutes per month	0.01
Number of sexual contacts a male individual has with a prostitute in a month	0.04

The results are significant at the 0.05 level (*), the 0.01 level (**) or the 0.001 level (***).

Uncertainty and sensitivity analyses of the epidemiological module

For the uncertainty and sensitivity analyses of the key parameters in the epidemiological module, we included the cofactor effect caused by co-infection. We sampled from the range of the cofactor values of the four infectious diseases included in the model, gonorrhea, syphilis, HSV-2 and malaria (Table S6).

The results from this analysis indicated that the variation generated by the uncertainty of the cofactors is higher than the variation generated by the uncertainty on the behavioral parameters (Table S7). The first empirical quartile indicated that at least in 75% of the simulations the HIV prevalence was higher than 10%. The sensitivity analysis indicated that infections with a short infectivity period but with high cofactor such as gonorrhea and syphilis, and the cofactor for infections present in the general population such as malaria, are the parameters contributing to the variation observed in the model (Table S8).

Table S6 - Parameters sampled and their range for the uncertainty analysis for the epidemiological module

Parameter	Range of uniform pdf	Reference
Amplificatory factor in HIV transmission		
Gonorrhea	[1.1-9]	[27-31]
Syphilis	[1.4-10]	[33-35]
Malaria	[1.1-1.6]	Derived from [26, 37]
Enhanced transmission of HIV in HSV-2 co-infected individual	[2-5]	[3, 13-14, 38-40]
Enhanced susceptibility to HIV in HSV-2 infected individual	[2-10]	[3, 13-14, 38-40]

Table S7 - Descriptive statistics from the uncertainty analysis for the epidemiological module

Descriptive Statistics	HIV prevalence
Mean	23.44
Median	22.83
Standard Deviation	7.35
25th percentile	17.02
95th percentile	32.80

Table S8 - Partial rank correlation coefficients of the parameters sampled from the epidemiological module

Parameter	PRCC
Amplificatory factor in HIV transmission	
Gonorrhea	0.42**
Syphilis	0.46**
Malaria	0.75***
Enhanced transmission to HIV in HSV-2 co-infected individual	0.04
Enhanced susceptibility to HIV in HSV-2 infected individual	0.29

The results are significant at the 0.05 level (*), the 0.01 level (**) or the 0.001 level (***).

Uncertainty and sensitivity analyses of the complete model

To explore the uncertainty and sensitivity of the complete model to the key behavioral and biological parameters, we performed an analysis that combined the ranges previously mentioned for both modules and the proportion of male population with circumcision [lower bound= 0.2; upper bound=0.5].

The combination of the uncertainty of the parameters of both modules increased the imprecision of the model. One important result from this analysis was that, in contrast with the uncertainty analysis for the epidemiologic module, the combination of both modules produced some combinations of behavioral parameters that did not allow the generation of an HIV epidemic. Thus, even with the inclusion of the cofactor effect, the results indicated that in 25% of the simulations an epidemic could not be produced (Table S9).

The results from the sensitivity analysis of the complete model were similar to the result of each module separately. The PRCCs showed that as in the behavioral module, the model was highly sensitive to the average duration of a casual relationship and the mean number of sexual contacts per month (Table S10). We observed that in simulations with an average duration of casual relationships of 2 months the HIV prevalence was less than 1%, whereas in simulations with an average duration of casual relationship longer than 4 months, the HIV prevalence was higher than 2%.

These results indicated the relevance of parameters that measure the risk of HIV transmission per partnership. Clearly, an increment in the duration of a relationship as well as the number of sexual contacts significantly increased the probability of HIV transmission and therefore, the HIV prevalence. Due to the methodological difficulties for measuring these parameters (especially the mean duration of a casual relationship), they are highly imprecise. Our model indicated however, the relevance of these parameters on epidemiological models.

Efforts focused on increasing the precision of their estimation will improve the accuracy of predictions from mathematical and computational models. On the other hand, the cofactors for infectious diseases present in the general population such as malaria and the enhanced HIV transmission caused by HSV-2 infection were the most influent epidemiological parameters.

Table S9 - Descriptive statistics from the uncertainty analysis for the complete module.

Descriptive Statistics	HIV prevalence
Mean	14.03
Median	7.12
Standard Deviation	16.02
25th percentile	0.23
95th percentile	45.06

Table S10 - Partial rank correlation coefficients of the parameters sampled from the complete module

Parameter	PRCC
Minimum age	-0.04
Maximum age	0.09
Number of sexual contacts per month	0.45***
Duration of casual relationship	0.79***
Proportion of married population	-0.10
Proportion of male population having sex with prostitutes per month	0.068
Circumcision	-0.17
Amplificatory factor in HIV transmission	
Gonorrhea	-0.03
Syphilis	-0.31
Malaria	0.23**
Enhanced transmission to HIV in HSV-2 co-infected individual	0.31**
Enhanced susceptibility to HIV in HSV-2 infected individual	0.10

The results are significant at the 0.05 level (*), the 0.01 level (**) or the 0.001 level (***).

Limitations of the model

Although the sexual network simulated allows for link dynamics, the degree distribution of the nodes and the node degree of each individual is maintained constant throughout the entire simulation. This assumption may be unrealistic, since the degree distribution of sexual networks on human populations might change over time. The node degree distribution of the network was estimated from data collected in 2003, and thus this degree distribution might not correspond to the node degree distribution of the sexual network in 1980. Intervention programs focused on preventing promiscuity and changes in behavior resulting from the HIV epidemic itself could decrease the number of sexual partners that a person has in a year. Consequently, the behavioral data for 2003 could underestimate the node degree distribution of the network at the beginning of the epidemic.

The static degree distribution also has an effect at an individual level. For simplification, and given the lack of data, we assume that each individual has the same node degree (number of sexual partners per year) during his/her entire sexual life. Although the degree distribution of the

network may be relatively stable for a long period of time, nodes (individuals) may change their degree over time. For example, an individual may start having many sexual partners; but marriage, possible infections with other STIs, and the HIV epidemic itself may generate a behavioral change in the individual, resulting in a lower number of sexual partners or even monogamy. This decision-making may be an important process in the network, where the inclusion of simulation tools, such as artificial intelligence, may be an interesting approach to simulating such behavioral changes.

We also assumed no control interventions for the other STIs and malaria, and therefore the prevalence of these infections remained constant over the simulation. This assumption may appear unrealistic since important efforts to control malaria and STIs in sub-Saharan Africa have been effective in reducing their prevalence; consequently, the effects of these diseases on HIV may have been decreasing in recent years.

Supplementary figures

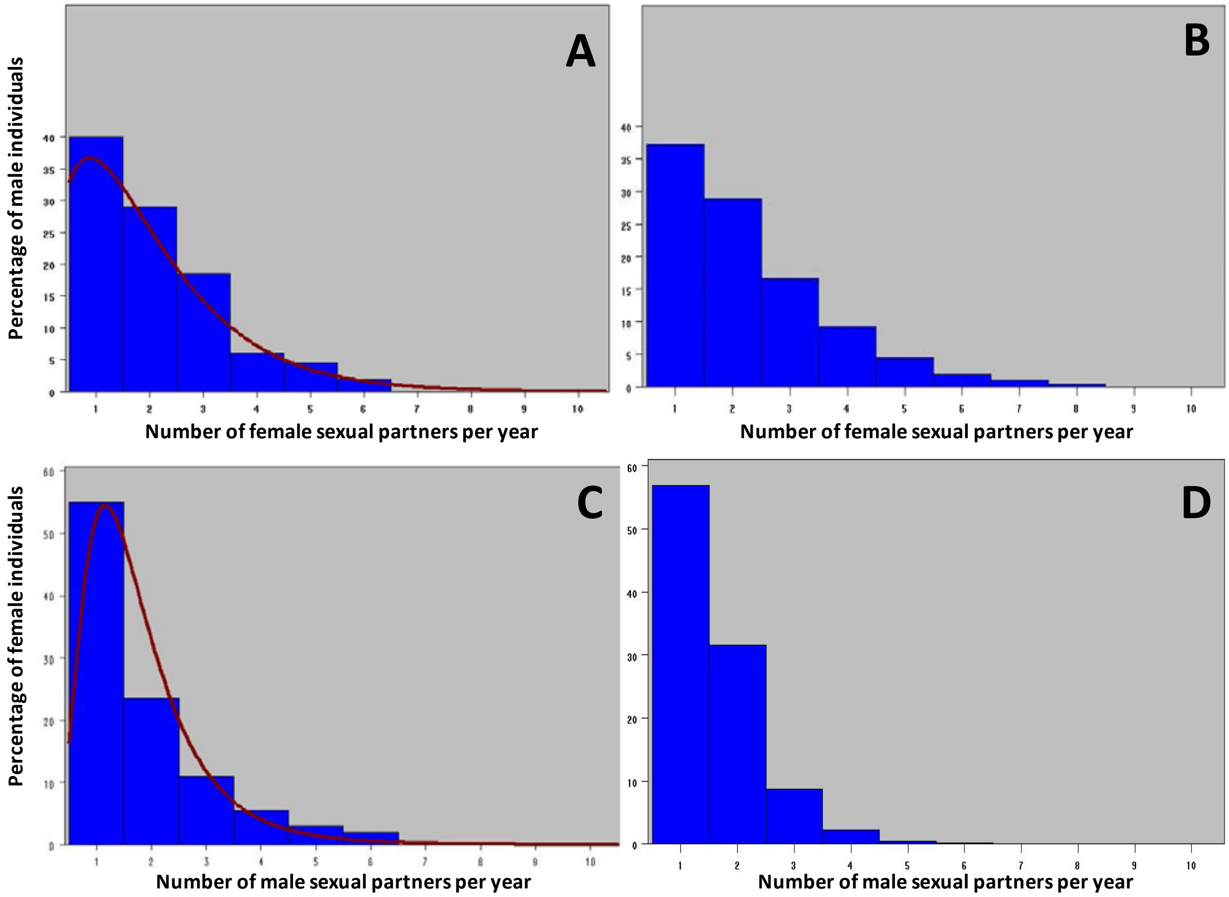

Figure S1 - Annual degree distribution of the number of sexual partners per year. Males: (A) Estimated for Malawi [1] (B) resulting from the simulations; and for females: (C) Estimated for Malawi [1] (D) resulted from the simulations. Red lines represent the fitted gamma distribution. The histograms represent the distribution of the data.

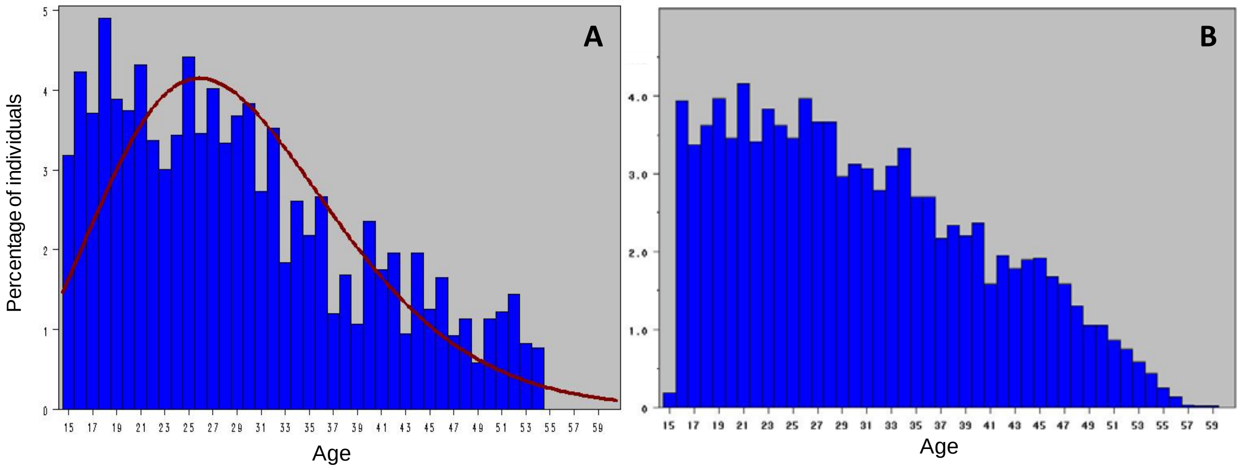

Figure S2 - Age distribution of males and females combined. (A) Age distribution of sexual active population estimated for Malawi in 2003 [2] and (B) Age distribution of sexual active population resulting from the simulation for 2003. Red lines represent the fitted gamma distribution. The histograms represent the distribution of the data.

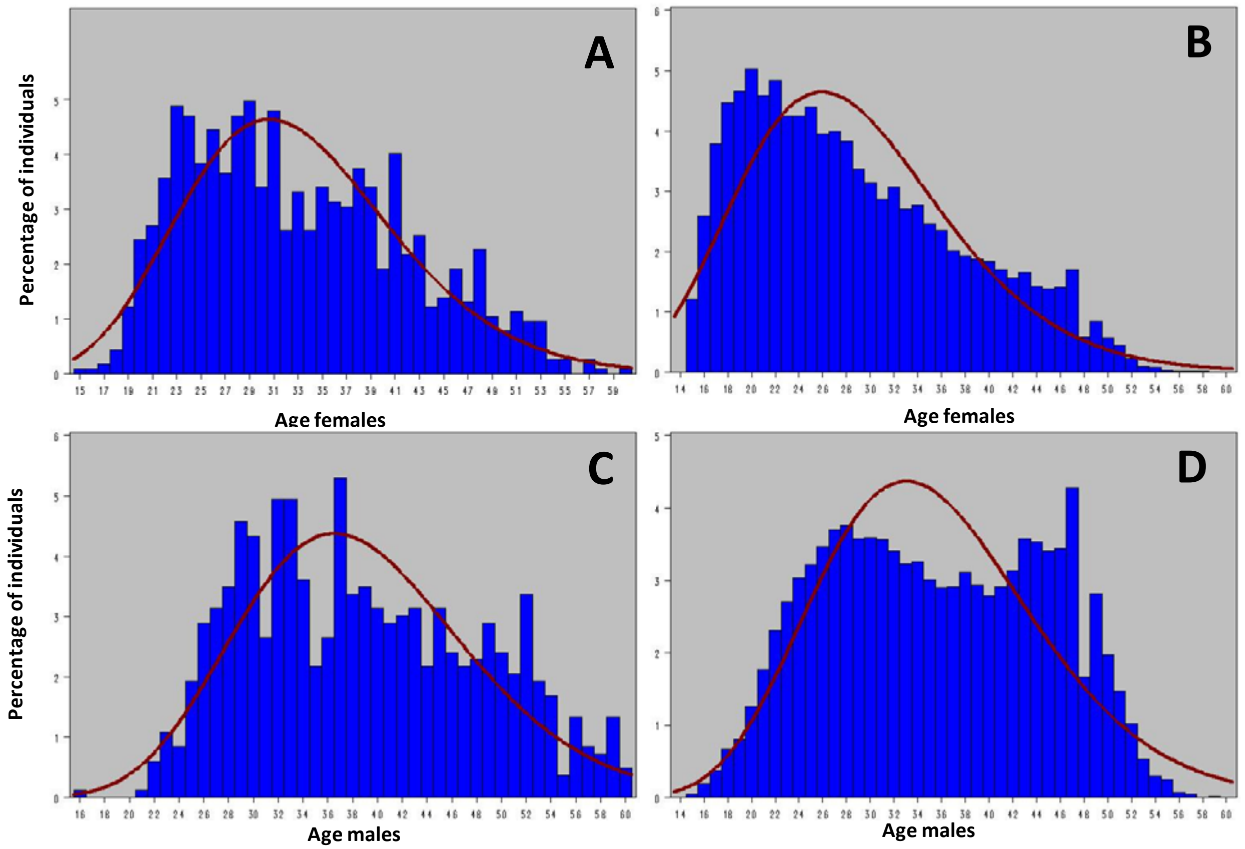

Figure S3 - Marriage distribution according to age. (A) Female age distribution estimated from Malawi in 2003 [2] and (B) the age distribution from the simulation estimated for 2003. (C) Male age distribution estimated from Malawi in 2003 [2] and (D) the age distribution from the simulation estimated for 2003. Red lines represent the fitted gamma distribution. The histograms represent the distribution of the data.

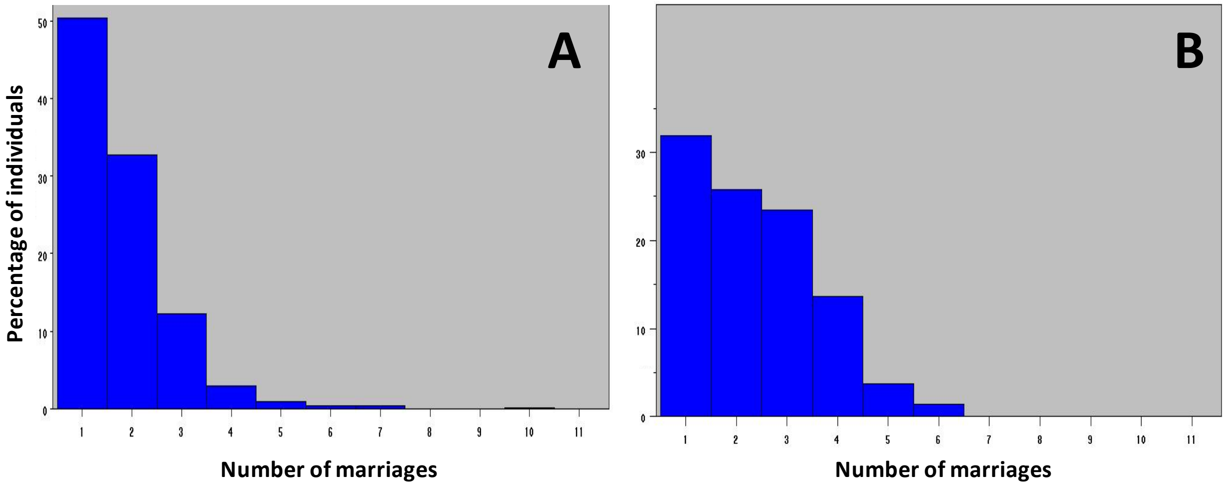

Figure S4 - Number of long-term relationships (marriages) during the entire sexual life of an individual male or female. (A) Derived from Malawi in 2003 [2] and (B) derivate from the simulations.

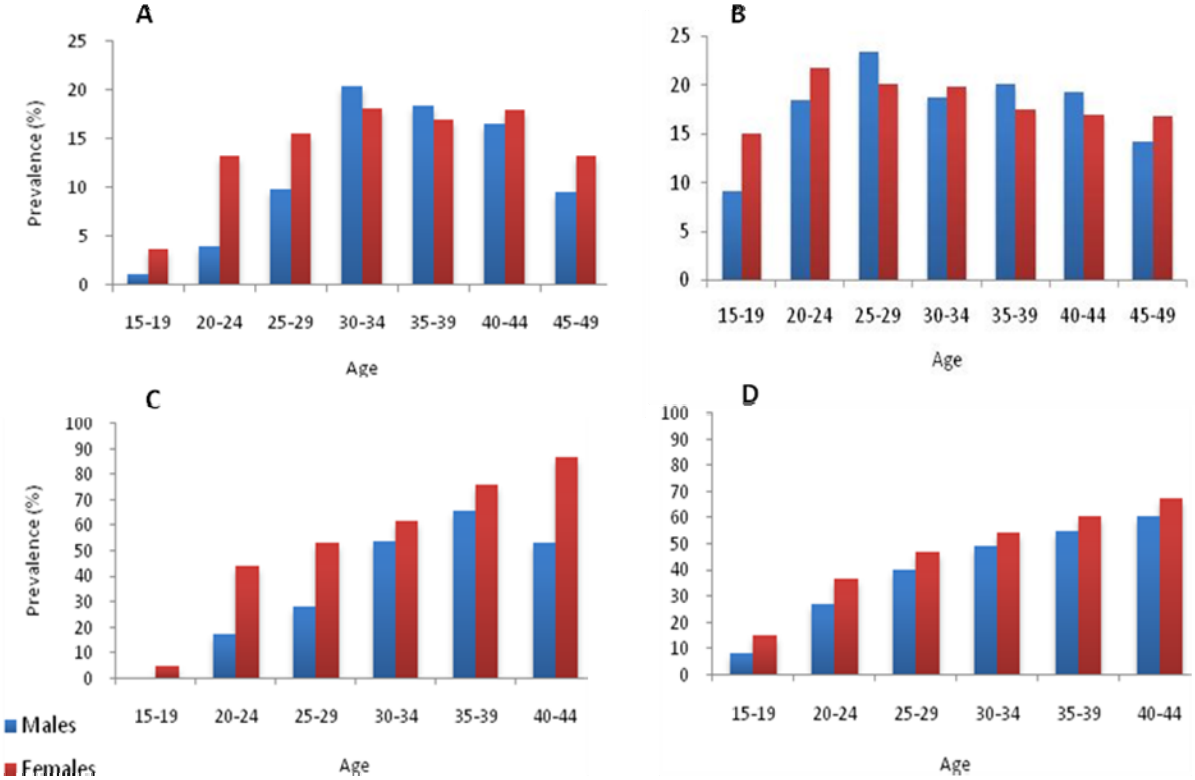

Figure S5 - Age-distribution of the HIV prevalence. (A) The observed data from Malawi in 2004 [46], (B) the resulting HIV prevalence distribution from Simulation 2 for the same year. (C) The observed age-distribution data from Malawi for HSV-2 in 2004 [46], and (D) the HSV-2 age-distribution prevalence from Simulation 2 for the same year.

References

1. University of Pennsylvania PSC: **Social network project, The Malawi Diffusion and Ideational Change Project (MDICP).** *Social network project, The Malawi Diffusion and Ideational Change Project (MDICP)*; 2003.

2. National Statistical Office ZM, and ORC Macro: **Malawi Demographic and Health Survey 2004.** *Malawi Demographic and Health Survey 2004*. City: Calverton, MD: NSO and ORC Macro.

3. Celum C, Levine R, Weaver M, Wald A: **Genital herpes and human immunodeficiency virus: double trouble.** *B World Health Organ* 2004, **82:** 447-453.

4. Gu W, Killeen GF, Mbogo CM, Regens JL, Githure JI, Beier JC: **An individual-based model of Plasmodium falciparum malaria transmission on the coast of Kenya.** *Trans Royal Soc Trop Med Hygiene* 2003, **97:**43-50.

5. Crampin AC, Floyd S, Glynn JR, Sibande F, Mulawa D, Nyondo A, Broadbent P, Bliss L, Ngwira B, Fine PE: **Long-term follow-up of HIV-positive and HIV-negative individuals in rural Malawi.** *AIDS* 2002, **16:**1545-1550.

6. Helleringer S, Kohler H: **Sexual network structure and the spread of HIV in Africa: evidence from Likoma Island, Malawi.** *AIDS* 2007, **21:**2323-2332.

7. Morison L, Weiss HA, Buvé A, Caraël M, Abega SC, Kaona F, Kanhonou L, Chege J, Hayes RJ; Study Group on Heterogeneity of HIV Epidemics in African Cities: **Commercial sex and the spread of HIV in four cities in sub-Saharan Africa.** *AIDS* 2001, **15:**S61-S69.

8. Ferry B, Caraël M, Buvé A, Auvert B, Laourou M, Kanhonou L, de Loenzien M, Akam E, Chege J, Kaona F; Study Group on Heterogeneity of HIV Epidemics in African Cities: **Comparison of key parameters of sexual behaviour in four African urban populations with different levels of HIV infection.** *AIDS* 2001, **15:**S41-S50.

9. Boily MC, Baggaley RF, Wang L, Masse B, White RG, Hayes RJ, Alary M: **Heterosexual risk of HIV-1 infection per sexual act: systematic review and meta-analysis of observational studies.** *Lancet Infec Dis* 2009, **9:**118-129.

10. Gray RH, Wawer MJ, Brookmeyer R, Sewankambo NK, Serwadda D, Wabwire-Mangen F, Lutalo T, Li X, vanCott T, Quinn TC; Rakai Project Team: **Probability of HIV-1 transmission per coital act in monogamous, heterosexual, HIV-1-discordant couples in Rakai, Uganda.** *Lancet* 2001, **357:**1149-1153.

11. Salomon JA, Murray CJ: **Modelling HIV/AIDS epidemics in sub-Saharan Africa using seroprevalence data from antenatal clinics.** *B World Health Organ* 2001, **79:**596-607.

12. Marion SA, Schecter MT: **Use of backcalculation for estimation of the probability of progression from HIV infection to AIDS.** *Statist Med* 1992, **12:**617-631.

13. Freeman EE, Orroth KK, White RG, Glynn JR, Bakker R, Boily MC, Habbema D, Buvé A, Hayes R: **Proportion of new HIV infections attributable to herpes simplex 2 increases over time: simulations of the changing role of sexually transmitted infections in sub-Saharan African HIV epidemics.** *Sex Transm Infect* 2007, **83:**i17-i24.

14. Abu-Raddad LJ, Magaret AS, Celum C, Wald A, Longini IM Jr, Self SG, Corey L: **Genital herpes has played a more important role than any other sexually transmitted infection in driving HIV prevalence in Africa.** *PLoS ONE* 2008, **3:**e2230. doi:2210.1371/journal.pone.0002230.

15. Orroth KK, Freeman EE, Bakker R, Buvé A, Glynn JR, Boily MC, White RG, Habbema JD, Hayes RJ: **Understanding differences between contrasting HIV epidemics in East and West Africa: results from a simulation model of the Four Cities Study.** *Sex Transm Infect* 2007, **83:**i5-16.

16. Buvé A, Weiss HA, Laga M, Van Dyck E, Musonda R, Zekeng L, Kahindo M, Anagonou S, Morison L, Robinson NJ, Hayes RJ; Study Group on Heterogeneity of HIV Epidemics in African Cities: **The epidemiology of gonorrhoea, chlamydial infection and syphilis in four African cities.** *AIDS* 2001, **15:**S79-S88

17. Ghani A, Swinton J. Garnett G: **The role of sexual partnership networks in the epidemiology of gonorrhea.** *Sex Transm Dis* 1998, **24:**45-56.

18. Pinkerton S, Abramson P: **Effectiveness of condoms in preventing HIV transmission.** *Soc Science Med* 1997, **44:**1303-1312.

19. Holmes K, Levine, R, Weaver M: **Effectiveness of condoms in preventing sexually transmitted infections.** *B World Health Organ* 2004, **82:**454-461.

20. Oxman G, Smolkowski K, Noell J: **Mathematical modeling of epidemic syphilis transmission. Implications for syphilis control programs.** *Sex Transm Dis* 1996, **23:**30-39.

21. Pourbohloul B, Reakart M, Brunham D, Robert C: **Impact of mass treatment on syphilis transmission: a mathematical modeling approach.** *Sex Transm Dis* 2003, **30:**297-305.

22. Doherty IA, Padian NS, Marlow C, Aral SO: **Determinants and consequences of sexual networks as they affect the spread of sexually transmitted infections.** *J Infect Dis* 2005, **191:**S42-S54.

23. Aron JL, May RM: **The population dynamics of malaria.** In *The Population dynamics of infectious diseases: theory and applications.* Edited by Anderson RM: Chapman and Hall 1982: 139-179.

24. Smith T, Maire N, Dietz K, Killeen GF, Vounatsou P, Molineaux L, Tanner M: **Relationship between the entomologic inoculation rate and the force of infection for plasmodium falciparum malaria.** *A Soc Trop Med Hygiene* 2006, **75:**11-18.

25. Anderson RM, May RM: *Infectious diseases of humans: dynamics and control.* Oxford: Oxford University Press; 1991.

26. Kublin JG, Patnaik P, Jere CS, Miller WC, Hoffman IF, Chimbiya N, Pendame R, Taylor TE, Molyneux ME: **Effect of Plasmodium falciparum malaria on concentration of HIV-1-RNA in the blood of adults in rural Malawi: a prospective cohort study.** *Lancet* 2005, **365**:233-240.

27. Laga M, Manoka A, Kivuvu M, Malele B, Tuliza M, Nzila N, Goeman J, Behets F, Batter V, Alary M, et al: **Non-ulcerative sexually transmitted diseases as risk factors for HIV-1 transmission in women: results from a cohort study.** *AIDS* 1993, **7:**95-102.

28. Kassler WJ, Zenilman JM, Erickson B, Fox R, Peterman TA, Hook EW 3rd: **Seroconversion in patients attending sexually transmitted disease clinics.** *AIDS* 1994, **8:**351-355.

29. Celentano DD, Nelson KE, Suprasert S, Eiumtrakul S, Tulvatana S, Kuntolbutra S, Akarasewi P, Matanasarawoot A, Wright NH, Sirisopana N, et al.: **Risk factors for HIV-1 seroconversion among young men in northern Thailand.** *JAMA* 1996, **275:**122-127.

30. Kapiga S, Lyamuya E, Lwihula G, Hunter D: **The incidence of HIV infection among women using family planning methods in Dar es Salaam, Tanzania.** *AIDS* 1998, **12:**75-84.

31. Galvin SR, Cohen, MS: **The role of sexually transmitted diseases in HIV transmission.** *Nature Rev Microb* 2004, **2:**33-42.

32. Sexton J, Garnett G, Rottingen J: **Metaanalysis and metaregression in interpreting study variability in the impact of sexually transmitted diseases on susceptibility to HIV infection.** *Sex Transm Dis* 2005, **32:**351-357.

33. Otten MW Jr, Zaidi AA, Peterman TA, Rolfs RT, Witte JJ: **High rate of HIV seroconversion among patients attending urban sexually transmitted disease clinics.** *AIDS* 1994, **8:**594-553.

34. Deschamps M, Pape J, Hafner A, Johnson W: **Heterosexual transmission of HIV in Haiti.** *Ann Intern Med* 1996, **125:**224-230.

35. Rakwar J, Lavreys L, Thompson ML, Jackson D, Bwayo J, Hassanali S, Mandaliya K, Ndinya-Achola J, Kreiss J: **Cofactors for the acquisition of HIV-1 among heterosexual men: prospective cohort study of trucking company workers in Kenya.** *AIDS* 1999, **12:**1699-1706.

36. Abu-Raddad LJ, Patnaik P, Kublin J: **Dual infection with HIV and malaria fuels the spread of both diseases in sub-Saharan Africa.** *Science* 2006, **314:**1603-1606.

37. Quinn T, Wawer M, Sewankambo N, Serwadda D, Li C,Wabwire-Mangen F, Meehan MO, Lutalo T, Gray RH for the Rakai Project Study Group: **Viral load and immune heterosexual transmission of human immunodeficiency virus type 1.** *N Engl J Med* 2000, **342:**921-929.

38. Blower S, Ma L: **Calculating the contribution of herpes simplex virus type 2 epidemics to increasing HIV incidence: treatment implications.** *Clinic Infect Dis* 2004, **39:**S240-S247.

39. Weiss HA, Buvé A, Robinson NJ, Van Dyck E, Kahindo M, Anagonou S, Musonda R, Zekeng L, Morison L, Caraël M, Laga M, Hayes RJ; Study Group on Heterogeneity of HIV Epidemics in African Cities: **The epidemiology of HSV-2 infection and its association with HIV infection in four urban African populations.** *AIDS* 2001, **15:**S97-S108.

40. Corey L, Wald A, Celum C, Quinn T: **The Effects of herpes simplex virus-2 on HIV-1 acquisition and transmission: a review of two overlapping epidemics.** *JAIDS* 2004, **35:**435-445.

41. Nzila N, Laga M, Thiam MA, Mayimona K, Edidi B, Van Dyck E, Behets F, Hassig S, Nelson A, Mokwa K, et al.: **HIV and other sexually transmitted diseases among female prostitutes in Kinshasa.** *AIDS* 1991, **5:**715-721.

42. Auvert B, Buvé A, Lagarde E, Kahindo M, Chege J, Rutenberg N, Musonda R, Laourou M, Akam E, Weiss HA; Study Group on the Heterogeneity of HIV Epidemics in African Cities: **Male circumcision and HIV infection in four cities in sub-Saharan Africa.** *AIDS* 2001, **15:**S31-S40.

43. Baeten JM, Richardson BA, Lavreys L, Rakwar JP, Mandaliya K, Bwayo JJ, Kreiss JK: **Female-to-male infectivity of HIV-1 among circumcised and uncircumcised Kenyan men.** *J Infect Dis* 2005, **191:**546–553.

44. Blower S, Dowlatabadi H: **Sensitivity and uncertanly analysis of complex models of disease transmission: an HIV model, as an example.** *Int Stat Rew* 1994, **62:**229-243.

45. Marino S, Hogue I, Ray C, Kirschner D: **A methodology for performing global uncertainty and sensitivity analysis in systems biology.** *J Theor Bio* 2008, **254:**178-196.

46. Glynn JR, Crampin AC, Ngwira BM, Ndhlovu R, Mwanyongo O, Fine PE: **Herpes simplex type 2 (HSV-2) trends in relation to the HIV epidemic in northern Malawi.** *Sex Transm Infect* 2008, **84:**356-360.
